# Investigation of the Developmental Requirements of Drosophila HP1 and Insulator Protein Partner, HIPP1

**DOI:** 10.1534/g3.118.200705

**Published:** 2018-12-04

**Authors:** Steve E. Glenn, Pamela K. Geyer

**Affiliations:** * Molecular Medicine Program; † Department of Biochemistry, University of Iowa, Iowa City, IA 52242

**Keywords:** Drosophila, Su(Hw), transcriptional regulation, chromatin insulator, HIPP1, HP1a, corepressor, CDYL

## Abstract

Drosophila Suppressor of Hairy-wing [Su(Hw)] is a multifunctional zinc finger DNA binding protein. Transcriptional regulation by Su(Hw) is essential in the ovary and testis, where Su(Hw) functions primarily as a repressor. Recently, the HP1a and Insulator Partner Protein 1 (HIPP1) was found to extensively co-localize with Su(Hw) and other insulator binding proteins in euchromatic regions of the genome, and with Heterochromatin Protein 1a (HP1a) in heterochromatic regions. As HIPP1 is the homolog of the human co-repressor Chromodomain Y-Like (CDYL), we tested its requirement in establishing transcriptional repression in flies. To this end, we generated multiple *Hipp1* null alleles and a tagged derivative of the endogenous gene (*Hipp1^GFP^*), using CRISPR mutagenesis. We show that HIPP1 is a widely expressed nuclear protein that is dispensable for viability, as well as female and male fertility. We find that HIPP1 and HP1a display minimum co-localization in interphase cells, and HP1a-dependent transcriptional repression of several reporter genes is HIPP1-independent, indicating that HIPP1 is not essential for HP1a-dependent heterochromatin formation. Despite Su(Hw) having a major role in promoting HIPP1 occupancy in euchromatin, we show that HIPP1 is dispensable for the transcriptional and insulator functions of Su(Hw), indicating that HIPP1 is not a critical Su(Hw) cofactor. Further studies are needed to clarify the role of HIPP1 in Drosophila development.

Development requires the precise temporal and spatial regulation of transcription. Central to these processes are DNA binding transcription factors (TF) that read the genome and execute chromatin changes to alter transcription. Multiple classes of DNA binding TFs exist, with Cys_2_His_2_ zinc finger (ZF) TFs representing the major class in metazoans (Enuameh *et al.* 2013; Lambert *et al.* 2018). Once bound, TFs impact transcription in multiple ways, including transcriptional activation and repression through targeted effects on promoters, as well as transcriptional insulation promoted by the formation of topological domains that shield promoters from inappropriate regulatory inputs. Although TFs were classically considered to have one effector function, much evidence suggests that individual TFs are multifunctional and demonstrate context-specific regulation (Reiter *et al.* 2017; Lambert *et al.* 2018). How single TFs achieve such multiplicity of effector function remains poorly understood.

Drosophila Suppressor of Hairy-wing [Su(Hw)] represents an exemplar multifunctional TF with insulator, activator and repressor functions (Geyer and Corces 1992; Roseman *et al.* 1993; Soshnev *et al.* 2008; Soshnev *et al.* 2013). Su(Hw) imparts transcriptional regulation using a twelve zinc finger domain to direct DNA binding (Spana *et al.* 1988). Insulator function of Su(Hw) depends upon binding to clusters of closely spaced binding sites, exemplified by binding to the cluster of twelve sites in the *gypsy* retrotransposon (Geyer *et al.* 1986; Geyer *et al.* 1988; Dorsett *et al.* 1989; Scott *et al.* 1999). In contrast, the activator and repressor functions of Su(Hw) are largely associated with standalone non-*gypsy* Su(Hw) binding sites [SBSs; (Soshnev *et al.* 2013)]. Of these transcriptional contributions, the Su(Hw) repressor function is the most prominent, based on findings that SBSs primarily localize within repressive ‘black’ chromatin (Filion *et al.* 2010) and nearby genes are generally derepressed upon Su(Hw) loss (Roy *et al.* 2010; Soshnev *et al.* 2013; Duan and Geyer 2018). The multiplicity of the Su(Hw) regulatory function has been linked to a “Su(Hw) code” (Baxley *et al.* 2017), wherein different combinations of Su(Hw) ZFs direct binding to SBSs carrying one of three sequence subclasses, each of which displays a distinct chromatin feature. These observations suggest that Su(Hw) DNA binding impacts cofactor recruitment, leading to context-specific transcriptional regulation.

Several cofactors have been identified that influence the Su(Hw) insulator function (Georgiev and Kozycina 1996; Gause *et al.* 2001; Pai *et al.* 2004; Kurshakova *et al.* 2007; King *et al.* 2014). Among these, the best characterized cofactors are the BTB/POZ domain proteins, Centrosomal Protein 190 kD and Modifier of mdg4 67.2 kD isoform (Mod67.2), two proteins required for enhancer blocking (Georgiev and Kozycina 1996; Pai *et al.* 2004) and a subunit of the SAGA histone acetyl transferase complex, Enhancer of yellow 2 (ENY2), that is needed for barrier function (Kurshakova *et al.* 2007). Strikingly, interaction of Su(Hw) with these insulator cofactors depends upon the ZF domain (Kurshakova *et al.* 2007; Melnikova *et al.* 2018). Notably, defects in ZFs 10 to 12 disrupt Su(Hw) association with CP190 and ENY2, concomitant with loss of Su(Hw) binding to the insulator subclass of SBSs and its insulator function. Together, these observations support that DNA binding influences the regulatory output of Su(Hw).

Su(Hw) cofactors required for its activator and repressor functions are unknown. HP1 and insulator partner protein (HIPP1, CG3680) is a newly identified factor that colocalizes with Su(Hw) (Alekseyenko *et al.* 2014; Rhee *et al.* 2014). In Drosophila S2 cells, BioTAP-XL mass spectrometry demonstrated that HIPP1 associates with multiple DNA binding insulator proteins (Alekseyenko *et al.* 2014), as well as Heterochromatin Protein 1a (HP1a). Of the insulator binding proteins (IBPs) studied, Su(Hw) has the strongest overlap with HIPP1 (56% of HIPP1 sites), with CCCTC-Binding factor (CTCF) representing the next common HIPP1 partner [19%, Figure 1; (Alekseyenko *et al.* 2014)]. HIPP1 also shows the strongest overlap with Su(Hw) relative to its other cofactors, associating with most (86%) SBSs and encompassing all sequence subclasses (Figure 1). This high degree of colocalization suggests that HIPP1 might contribute to Su(Hw) regulation.

**Figure 1:**
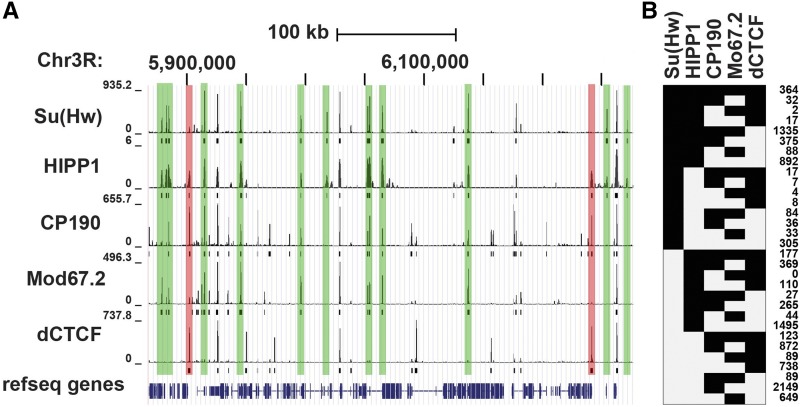
HIPP1 is the major Su(Hw) cofactor. A. Shown is a view from the UCSC Genome Browser of a representative 418 kb region of chromosome 3R. ChIP-seq tracks (top) and called peaks (bottom) are shown for Su(Hw), HIPP1, CP190, Mod67.2, dCTCF using datasets from S2 cells (Soshnev *et al.* 2012; Ong *et al.* 2013; Alekseyenko *et al.* 2014). B. Summary of the frequency of co-localization of insulator proteins at individual genomic regions using data shown in A. The number of occurrences of the particular combination of insulator proteins is indicated at the right.

HIPP1 is the fly homolog of vertebrate Chromodomain Y-like (CDYL) proteins, a family of transcriptional co-repressors (Caron *et al.* 2003; Mulligan *et al.* 2008). CDYL proteins carry an amino-terminal chromodomain that binds methylated H3K9 and H3K27 (Franz *et al.* 2009) and a carboxyl-terminal domain that displays homology with lipid-metabolizing enzymes of the crotonase superfamily (Lahn *et al.* 2002; Caron *et al.* 2003; Wu *et al.* 2009; Zhang *et al.* 2011). Biochemical analyses established that the crotonase domain functions both as a protein-protein interaction platform that recruits co-repressors such as histone deacetylases (Caron *et al.* 2003; Escamilla-Del-Arenal *et al.* 2013), as well as a crotonyl-CoA hydratase that negatively regulates histone lysine crotonylation (Liu *et al.* 2017), a histone modification associated with active transcription. These findings implicated HIPP1 as a candidate Su(Hw)-dependent co-repressor.

Here, we study the function of HIPP1. To this end, we made multiple *Hipp1* alleles, including tagging the endogenous gene to generate *Hipp1^GFP^*. Using these tools, we investigated the developmental expression and functional contributions of HIPP1. We find that HIPP1 is a widely expressed nuclear protein. Surprisingly, our immunohistochemical analyses uncovered limited co-localization between HIPP1 and HP1a, and our genetic studies revealed that HIPP1 loss does not reverse HP1a-dependent transcriptional repression of several reporter genes, indicating that HIPP1 is not essential for HP1a-dependent heterochromatin formation. Further, we demonstrate that *Hipp1* null mutants are viable, as well as female and male fertile. Despite Su(Hw)-dependent HIPP1 localization at SBSs, we found that HIPP1 loss does not compromise the transcriptional or insulator functions of Su(Hw), indicating that HIPP1 is a non-essential Su(Hw) cofactor. Further studies are needed to resolve the role of HIPP1 in Drosophila development.

## Materials and Methods

### Drosophila stocks and culture conditions

All Drosophila stocks were raised on standard cornmeal/agar medium at 22°. Two *su(Hw)^+/+^* strains were used in this study, including (1) *y^1^w^1118^* and (2) Canton-S (Bloomington Stock Center, BL1). Three *su(Hw)* null alleles were used, including (1) *su(Hw)^v^* that carries a ∼1.7 kb deletion encompassing the *su(Hw)* and *RpII15* promoters (Harrison *et al.* 1992), *(*2) *su(Hw)^2^* that carries an insertion of an ∼1.3 kb element into the first intron of the *su(Hw)* gene (Parkhurst *et al.* 1988), and (3) *su(Hw)^Pb^* that carries an insertion of a *white* marked *piggyBac* element into the second exon of the *su(Hw)* gene [*su(Hw)^e04061^* in Flybase]. Other stocks used include *Su(var)2-5^04^* and *Su(var)3-9^06^* provided by Lori Wallrath (U of Iowa), three *SUPor P* lines (KV108, KV135, KV590) that are insertions into heterochromatic regions that were provided by Keith Maggert (University of Arizona) and Gary Karpen [U of California, Berkeley; (Konev *et al.* 2003)] and *T(2;3)Sb^V^* (BL 878). *Su(var)2-5^04^* results from a point mutation that changes lysine 169 to a stop codon (Eissenberg *et al.* 1992). *Su(var)3-9^06^* results from a ∼6 kb DNA insertion that blocks transcript accumulation (Westphal and Reuter 2002). *SUPor P* is a composite *P* transposon that contains the *mini-white* gene and associated eye enhancer positioned between two *gypsy* insulators and the intronless *yellow* gene that carries wing and body enhancers. When *SUPor P* is inserted into heterochromatic regions, variegation of *yellow* and/or *white* expression is observed (Roseman *et al.* 1995; Konev *et al.* 2003). *T(2;3)Sb^V^* results from an inversion plus translocation of the *Sb^1^* mutation, positioning *Sb^1^* adjacent to the centric heterochromatin in the right arm of chromosome 2 (Beaton *et al.* 1988).

### Generation of Hipp1 alleles

CRISPR was used to generate multiple *Hipp1* alleles, using methods outlined in (Bier *et al.* 2018). Small deletions were generated by embryo injection of single guide RNA expression plasmids, made from pCFD3 (Addgene plasmid 49410). Injected embryos expressed germline Cas9 (*yw*; *nos-Cas9[II-attP40]*, Bestgene). Putative mutants were screened using a PCR-based restriction enzyme assay, with candidates confirmed using genomic sequencing. Five small deletion alleles were generated: *1G3*, *1G5*, *2G4*, *3G6*, and *3G10*. The large deletion allele *Hipp1^Δ37^* was generated by injection of a pair of guide RNA plasmids. A summary of the molecular details of *Hipp1* alleles can be found in Fig. S1 and Table S1, with the list of primers used for PCR analyses found in Table S2. Finally, a *Hipp1* replacement allele was generated that swapped sequences -314 and +3913 of *Hipp1* with DsRed (*Hipp1^ΔDsR^*). In this mutagenesis, a guide RNA plasmid was co-injected with a pDsRed-attP (Addgene 51019) derivative that carried 1 kb of upstream and downstream *Hipp1* sequences relative to the guide RNA cutting sites (Fig. S1).

*Hipp1^GFP^* was generated using the scarless tagging method described in (Bier *et al.* 2018). Briefly, gRNA expression plasmids targeting positions +3179 and +3240 of *Hipp1* were co-injected with a template plasmid containing homology arms flanking the GFP coding sequence cloned adjacent to a piggyBac transposon that contained a DsRed expression construct (pHD-sfGFP-ScarlessDsRed, DGRC #1365). Primers used to clone the homology arms included a synonymous G to C mutation at +3174 (Ala to Ala) and a G to C change at +3236 in the 3′ untranslated region to eliminate homology to the PAM sequences in the endogenous *Hipp1* gene. Template plasmid and gRNA expression plasmids were co-injected by Bestgene (Stock name: *yw;nos-Cas9[II-attP40]*). DsRed positive flies were crossed to a piggyBac transposase expressing line (Bloomington stock #8285) to excise DsRed, resulting in an in-frame fusion of the *Hipp1* coding and GFP coding sequences. Successful generation of *Hipp1^GFP^* was confirmed by sequencing.

### Generation of HIPP1 antibody and western analysis

Peptide Specialty Laboratories (Heidelberg Germany) generated two polyclonal guinea pig HIPP1 antibodies. The two peptide antigens used included amino acids 570 to 585 (TSARKPRASDSWDYVY) and 599 to 620 (RSNSSYSSNASVSRNSLDNRPG). Antibodies were affinity purified using a bacterially purified HIS-tagged HIPP1 protein carrying amino acids 454 to 630.

Western analysis of HIPP1 protein was performed using ovary extracts obtained from 1- to 3-day-old females and testes extracts from <1 day-old males. Western blots were incubated with affinity purified HIPP1 antibody (1:100) that was pre-incubated with >100-fold excess of peptide 2 for one hour to reduce background. HIPP1 was detected using horse radish peroxidase (HRP) conjugated second antibodies (1:20,000; Jackson 706-035-148) and analyzed using Advansta WesternBright Quantum chemiluminescent kit (K-12042-D20). Blots were re-probed with anti-alpha-tubulin antibody (1:20,000; Sigma T5168) to serve as a loading control. Western analysis of histone crotonylation was performed using ovary extracts obtained from 1- to 3-day old females and testis extracts obtained from <1-day-old males. Membranes were incubated with rabbit pan α-crotonyl-lysine (panKcr) antibody (1:2,000; PTM-501) or rabbit α-H3 antibody (1:2,000; Abcam 791-100). Proteins were detected using secondary HRP antibodies (1:20,000; BioRad 172-1019) and detected using ECL detection reagents (GE Healthcare, RPN2106).

### Immunohistochemical analyses

Larval imaginal discs and the central nervous system, as well as adult ovaries (1< day-old) and testes (three-day-old) were dissected into PBS and stained as described previously (Baxley *et al.* 2011; Duan and Geyer 2018). Primary antibodies were diluted in 5% BSA, 0.3% TritonX-100 in PBS and incubated with tissues overnight at 4°. Following washes, tissues were stained with 1 μg/ml DAPI (ThermoFisher Scientific) and mounted in ProLong Diamond Antifade Mountant (Invitrogen P36961). Antibodies include polyclonal goat α-Su(Hw) at 1:300 dilution (Baxley *et al.* 2011), rabbit polyclonal α-GFP (Life Technologies A11122) at 1:1,000, mouse monoclonal α-HP1a (DSHB C1A9) at 1:200 and mouse α-pan polyglycylated tubulin (Millipore AXO49) at 1:500. Secondary antibodies include donkey α-rabbit AF488 (Invitrogen A21206), donkey α-goat AF568 (Life Technologies A11057), donkey α-rabbit AF568 (Invitrogen A10042), donkey α-goat 488 (Life Technologies A11055), and donkey α-mouse 647 (Invitrogen A31571). Secondary antibodies were used at a 1:500 dilution. Actin was stained with Texas Red-X phalloidin (Life Technologies T7471) at 1:500. All images were collected on a Zeiss LSM 710 Confocal Microscope and assembled with Adobe Illustrator.

### Fecundity and Position Effect Variegation (PEV) assays

Female fecundity was measured by mating eight females of each genotype to four Canton-S males in bottles that were capped with orange juice plates spread with yeast paste. Every 24 hr, orange juice plates were replaced, and the eggs that were laid were counted. Male fertility was assayed using sperm exhaustion assay as described in (Barton *et al.* 2016; Duan and Geyer 2018). Briefly, one-day-old males were mated to three virgin females for 3 days, at the end of this period males were transferred to new vials with three fresh virgin females. This mating scheme was repeated for 15 days. Males were scored as fertile if they produced at least five progeny from a three day mating period.

To determine effects of loss of HIPP1 on heterochromatin structure, we tested the ability of *Hipp1* mutants to modify variegation of several variegating alleles. First, we studied PEV of the *Sb^1^* allele in the context of *T(2:3)Sb^v^* (Dietz *et al.* 2015). These flies carry a chromosomal translocation that places the dominant *Sb^1^* mutation adjacent to centric heterochromatin of the second chromosome. We crossed wild type (*y w* ), *Su(var)2-5^04/+^*, *Su(var)3-9^06/06^* and *Hipp1^−/−^* females to *T(2:3)Sb^V^ / TM3 [Ser]* males and quantified the length of six bristles on the thorax of adult females. Increased levels of heterochromatin inactivate the dominant *Sb^1^* mutation, restoring bristle length from short (stubble) to long. As such, higher levels of heterochromatin are associated with higher frequencies of long bristles. Second, we studied PEV of multiple *SUPor P* lines, including the KV00590, insertion site at *Y*:3472914, KV108 insertion site on the *Y* and KV135, insertion site at *Chr2R*:1224899 (Konev *et al.* 2003; Swenson *et al.* 2016). All of these *SUPor P* lines are sensitive to levels of HP1a. We crossed homozygous *Hipp1^1G3^*, *Hipp1^ΔDsR^* and *Hipp1^Δ37^* mutant females to males from each reporter line and determined pigmentation levels in newly eclosed progeny. Recessive effects of *Hipp1* on PEV were determined by crossing males of each reporter line to *Hipp1^ΔDsR^* females and then crossing the resulting male progeny to homozygous *Hipp1^Δ37^* females, thereby generating *Hipp1^ΔDsR/ Δ37^* males.

### Quantitative PCR analysis of gene expression

RNA was isolated from either 75 pairs of <1 day-old ovaries or 100 pairs of three-day old testes per biological replicate as described previously (Soshnev *et al.* 2008). Genomic DNA was removed using Invitrogen DNase treatment and removal kit (Cat# AM1906). Generation of cDNA was done using Applied Biosystems Reverse transcription kit (Cat# 4368814). Expression levels were normalized to *RpL32* and to one of the replicates of Canton-S RNA. Primer sequences are listed in Table S3.

### Chromatin Immunoprecipitation (ChIP)

Chromosome association of HIPP1-GFP and Su(Hw) was tested using ChIP from 100-150 1< day-old ovaries per biological replicate. ChIP for HIPP1-GFP represented a modification of our standard protocol (Baxley *et al.* 2011), wherein chromatin was cross-linked with 3.0% formaldehyde for 30 min (Alekseyenko *et al.* 2014), as compared to 1.8% formaldehyde for 10 min. As a negative control for HIPP1-GFP ChIP, GFP antibodies were used to ChIP chromatin from wild type (Canton-S ovary chromatin). In these studies, all sites showed less than 0.3% input (data not shown), demonstrating the specificity of the GFP ChIP. Antibodies used in ChIP experiments include rabbit polyclonal α-GFP (Abcam Ab290) and guinea pig polyclonal α-Su(Hw) (Baxley *et al.* 2011). Immunoprecipitated DNA was quantified using quantitative real time PCR (qPCR) with SYBR green (Bio-Rad Cat# 170-8882). Analyses were performed on at least two biological replicates. Statistical analysis was performed using PRISM. Primers sequences are listed in Table S4.

### Data availability

Strains and plasmids are available upon request. Supplemental material available at Figshare: https://doi.org/10.25387/g3.7399337.

## Results and Discussion

### HIPP1 carries a conserved crotonase domain

**Figure 2:**
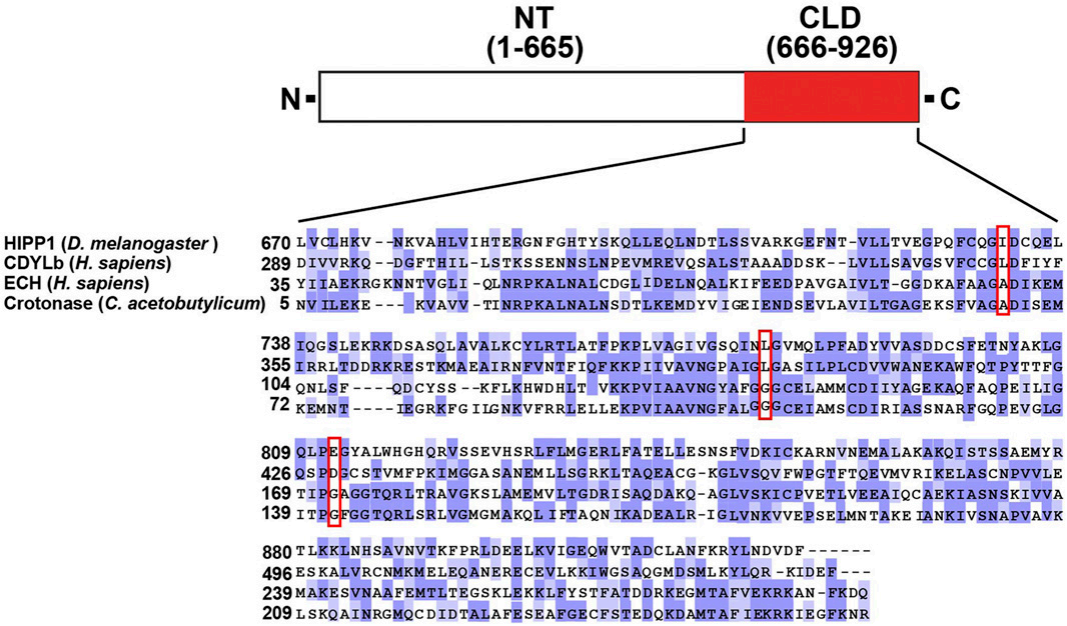
HIPP1 is the fly homolog of human CDYL. Shown is a diagram of the HIPP1 protein, highlighting the C-terminal crotonase-like domain (amino acids 666 to 926, CLD, red). Below the protein diagram is an alignment of the HIPP1 CLD with crotonase domains from human CDYLb, Enoyl-coA hydratase, and bacterial Crotonase. Shading indicates identical (dark purple) and similar (light purple, PAM250 matrix score >0.5) residues. Red boxes show the location of the three structural amino acid residues (red boxes) that are predicted to form the oxyanion hole in CDYL (Wu *et al.* 2009).

Reciprocal affinity purifications identified the fly homolog of human CDYL as a Su(Hw) cofactor (Alekseyenko *et al.* 2014; Rhee *et al.* 2014). Three variants of human CDYL have been identified (Franz *et al.* 2009), of which only CDYL1b carries a functional chromodomain. For the other variants, one has an N-terminal extension that inactivates the chromodomain (CDYL1a) and the second lacks the chromodomain (CDYL1c). To understand the relationship of HIPP1 to human CDYL, we defined the structural conservation of HIPP1 and CDYLb (Figure 2). Alignment of the amino termini of these proteins provided evidence of an extended amino terminal region that showed signs of a degenerated chromodomain in HIPP1, wherein only a short region of homology was found that included two of the three aromatic cage residues essential for binding methylated lysine [data not shown, (Jacobs and Khorasanizadeh 2002)]. Based on these observations, we conclude that fly HIPP1 lacks a functional chromodomain (data not shown), a conclusion that is reinforced by alignment with other drosophilid HIPP1 homologs (data not shown). These data imply that HIPP1 is most similar to the non-chromodomain CDYL variant, CDYL1a. Alignment of the HIPP1 carboxyl termini with CDYLb identifies a crotonase-like fold domain (CLD; Figure 2). In the crotonase superfamily, this domain carries the active site of the enzyme, wherein conserved structural elements preserve the formation of an “oxyanion hole” that is needed for stabilization of an enolate anion intermediate derived from an acyl-CoA substrate. The crystal structure of CDYL identified L403, L452 and D483 as the critical residues forming the oxyanion hole (Wu *et al.* 2009). These residues are conserved in HIPP1 (732I, 780L and 812E) and are invariant in other drosophilid HIPP1 proteins (data not shown). Our findings suggest that HIPP1 carries a functional crotonase domain.

### Hipp1 is a non-essential gene

**Figure 3:**
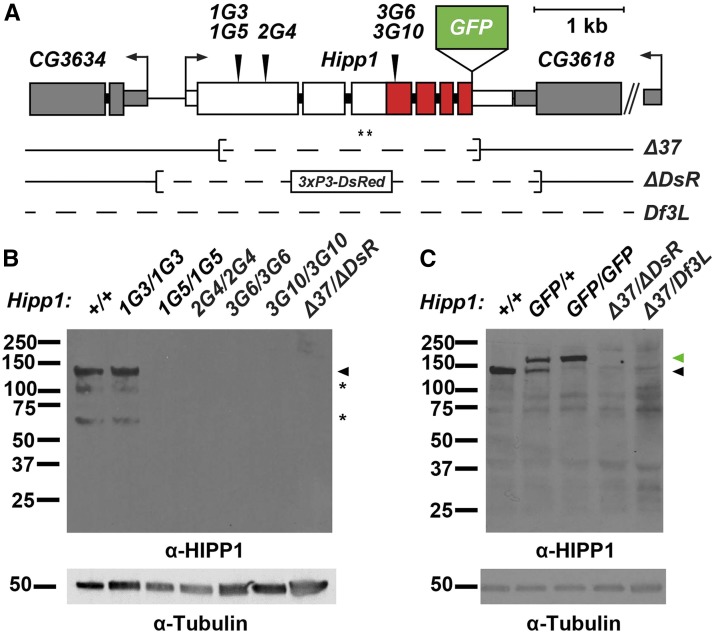
Structure of the *Hipp1* locus. A. Shown is the structure of the *Hipp1* gene, with exons (large rectangles) colored to indicate the positions of the crotonase-like domain (CLD, red) and bent arrows to show directions of transcription. Structures of the neighboring *CG3634* and *CG3618* genes are indicated in gray. Inverted triangles above the *Hipp1* gene indicate the locations of the small CRISPR generated deletions (*1G3*, *1G5*, *2G4*, *3G6* and *3G10)*, whereas the locations of the large deletions *(Δ37* and *Δ DsR*) are shown below the gene. The position of insertion of the GFP coding region is shown (raised green rectangle). Asterisks indicate the location of the peptide epitopes recognized by the HIPP1 antibody. B., C. Western blot of protein extracts obtained from ovaries dissected from wild type (+/+, *Canton S*) or *Hipp1* mutant females of the indicated genotype. Blots were probed with the HIPP1 antibodies, using antibodies against alpha-Tubulin as a loading control. Positions of full-length proteins are shown by black and green arrowheads, indicating HIPP1 and HIPP1-GFP, respectively. Asterisks indicate positions of degradation products.

To understand the HIPP1 function, we generated multiple mutant *Hipp1* alleles using CRISPR. These included small and large deletions within the *Hipp1* coding region, which were confirmed by PCR and sequence analysis (Figure 3A, Fig. S1, Table S1). Western blots of ovary extracts assessed effects of these CRISPR-induced indels on protein production. Of the *Hipp1* alleles with small deletions, four (*1G5*, *2G4*, *3G6*, *3G10*) were predicted to cause premature termination of the encoded protein, whereas the fifth mutant (*1G3*) was predicted to remove two amino acids (Table S1). Indeed, we found that the four putative premature termination mutants failed to accumulate any HIPP1 protein, while the fifth generated a full-length protein (Figure 3B). No HIPP1 protein was detected in flies carrying the two large deletion alleles (*Δ37* and *Δ DsR*). In total, six *Hipp1* null alleles were generated.

Once *Hipp1* mutants were available, we defined effects of HIPP1 loss on viability. To this end, we crossed *Hipp1^-^/ TM6c* males and females and determined the number of *Hipp1^-/+^* (*TM6c*, *Sb*) *vs.* *Hipp1^−/−^* (non-*TM6c*, *Sb*) progeny. We found that *Hipp1* adults were obtained at or near the expected number, with these adults displaying normal morphology (Table 1). We conclude HIPP1 is not essential for Drosophila development. Strikingly, these findings contrast those for CDYL, wherein the knockout mouse is inviable (Wan *et al.* 2013).

**Table 1:** Percent viability of *HIPP1* mutants

	Allele transmitted by female					
Allele transmitted by male	*Δ37/TM6c*		*ΔDsR/TM6c*		*Df(3L)_b_/TM6c*	
	% viable*^a^*	# of *TM6c*	% viable*^a^*	# of *TM6c*	% viable*^a^*	# of *TM6c*
*Δ37/TM6c*	ND	ND	112	251	111	202
*Δ DsR/TM6c*	84	319	ND	ND	92	218
*Df3L/TM6c*	84	119	142	257	ND	ND
*GFP/TM6c*	88	57	80	383	116	372

a Percent viability was determined by dividing the total number of non-balancer progeny obtained by half of the total number of *TM6c* progeny, multiplied by 100.

b *Df(3L)* refers to *Df(3L)BSC452* that carries a 196 kb deletion that includes *HIPP1* and 28 other genes. Viability of homozygous mutants was not defined (ND).

### HIPP1 is broadly expressed during development

To understand possible developmental roles, we defined the tissue distribution of HIPP1. As our peptide antibodies did not work in immunohistochemistry, we used CRISPR technology to engineer the endogenous gene to encode HIPP1-GFP (Figure 3A), choosing a carboxy-terminal tag based on previous studies of CDYL (Escamilla-Del-Arenal *et al.* 2013). Western analysis of proteins obtained from *Hipp1^GFP/+^* females demonstrated that HIPP1-GFP was stably produced at wild type levels (Figure 3C). Furthermore, genetic analyses demonstrated that *Hipp1^GFP^* adults are produced at wild type levels (Table 1). We conclude that HIPP1-GFP serves as a faithful reporter of HIPP1.

**Figure 4:**
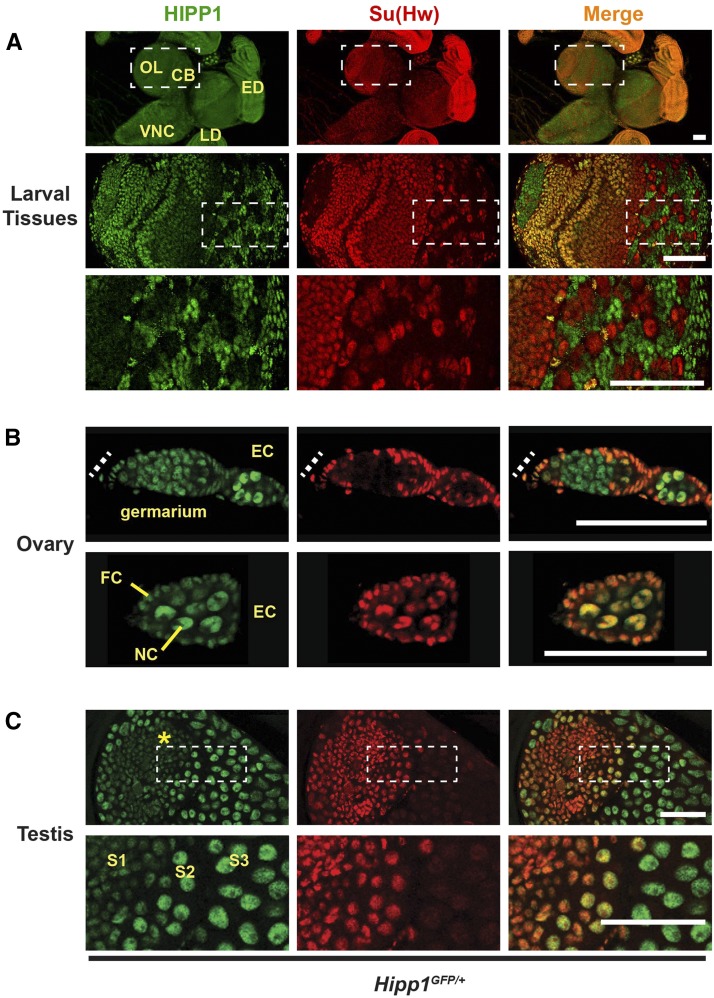
HIPP1 is a globally expressed nuclear protein. A-C. Confocal images of tissues dissected from *Hipp1^GFP/+^* and stained with antibodies against GFP (HIPP1, green) and Su(Hw) (red), with the merged image at the right. A. *Top panels:* Representative images of tissues dissected from third instar larvae, showing neuronal tissues of the central brain (CB), optic lobe (OL), ventral nerve cord (VNC), as well as non-neuronal tissues (eye disc, ED; leg disc, LD). *Bottom panels*: Magnification of boxed region of the central brain isolated from a *Hipp1^GFP/+^* wandering third instar larva. This section reveals that some cell types express Su(Hw), but not HIPP1. Scale bars, 50 μm. B. *Top panels:* Image of a germarium, with the position of the somatic niche shown as a dashed line. *Bottom panels:* an early stage egg chamber (EC, *bottom*) that contains differentiated germ cells (nurse cells, NC) surrounded by somatic follicle cells (FCs). Scale bars, 20 µm. C. *Top panels:* Image of a testis that shows the somatic niche (hub, asterisk) and developing germ cell cysts. *Bottom panels*: Magnification of the boxed region to highlight the transition between Su(Hw) positive spermatocytes (stages S1 to S2) and Su(Hw) absent spermatocytes (S3). HIPP1 expression is stronger in mid-to-late stage spermatocytes. Scale bars, 50 µm.

We used *Hipp1^GFP^* individuals to determine whether HIPP1 and Su(Hw) are commonly co-expressed. Larval tissues were examined first. These experiments revealed that HIPP1 is nuclear enriched (Figure 4), a finding that contrasts with the localization of other members of the crotonase family that are found in the cytoplasm, specifically in peroxisomes and mitochondria (Furuta *et al.* 1980; Geisbrecht *et al.* 1999). Expression of HIPP1 extensively overlaps with that of Su(Hw) in larval tissues, with the exception of brain and the ventral nerve cord (Figure 4A). In these neuronal tissues, HIPP1-GFP is largely present, whereas Su(Hw) is largely absent. Even so, the optic lobe and central brain carry clusters of cells that express only Su(Hw), and not HIPP1 (Figure 4A). These studies show that HIPP1 and Su(Hw) extensively co-localize but are not obligate partners.

We determined the spatial localization of HIPP1-GFP in the ovary, an adult tissue that requires Su(Hw) (Baxley *et al.* 2011; Duan and Geyer 2018). Drosophila ovaries are divided into ovarioles that carry an organized developmental program of advancing stages of oocyte maturation (Bastock and St Johnston 2008). At the anterior end of an ovariole is the germarium, a specialized structure that contains somatic cells that comprise the stem cell niche and two to three germline stem cells (GSCs). Upon GSC division, differentiating germ cells undergo four incomplete mitotic divisions to form a sixteen-cell cyst called an egg chamber. Continued germ cell differentiation produces an egg chamber with fifteen polyploid nurse cells, one diploid oocyte and a surrounding layer of somatic follicle cells. In the absence of Su(Hw), oogenesis is blocked, due to complete apoptosis of mid-stage egg chambers. This defect results from loss of transcriptional regulation in both somatic and germ cells (Soshnev *et al.* 2013). We co-stained ovaries dissected from *Hipp1^GFP^* females with GFP and Su(Hw) antibodies to define their extent of co-localization. These studies revealed that HIPP1-GFP is present in all somatic and germ cells (Figure 4B), even in the mitotically active region of the germarium that lacks Su(Hw). In later stages of oogenesis, HIPP1-GFP localization parallels that of Su(Hw), being found on nurse cell chromosomes but excluded from nuclear regions that contain the nucleolus (Figure 4B). These studies reveal extensive co-localization of HIPP1 and Su(Hw) in the ovary.

Su(Hw) is also required in testes for sperm development (Duan and Geyer 2018). For this reason, we examined HIPP1 expression in the testis. The Drosophila testis has a single stem cell niche, called the hub, that supports two stem cell populations, GSCs and cyst stem cells. Spermatogenesis begins upon asymmetric division of both stem cell populations to form a differentiation unit of somatic cyst cells and germ cells. Subsequent mitotic and meiotic divisions of the germ cells produce 64 spermatids that differentiate into sperm. In the absence of Su(Hw), males demonstrate an age-dependent block in late stages of spermatogenesis, resulting in a failure to produce sperm. These defects result from a loss of Su(Hw) in somatic cells of the testis (Duan and Geyer 2018). Expression of HIPP1 and Su(Hw) was determined by co-staining testes isolated from *Hipp1^GFP^* males with antibodies against GFP and Su(Hw). We found low levels of HIPP1-GFP in all somatic cells (Figure 4C, data not shown). In contrast, HIPP1-GFP and Su(Hw) are produced in a complementary pattern in germ cells. Early spermatocytes have high levels of Su(Hw) that diminish as they develop, while HIPP1-GFP levels start out low and then increase at the time Su(Hw) declines (Figure 4C). Although Su(Hw) and HIPP1 overlap is less extensive as compared to other non-neuronal tissues, HIPP1 is present in the cell type where Su(Hw) function is essential for male fertility (Duan and Geyer 2018). These immunohistochemical studies reveal that HIPP1 and Su(Hw) co-localize in the testis where Su(Hw) regulation is required.

### HIPP1 has a limited partnership with HP1a

**Figure 5:**
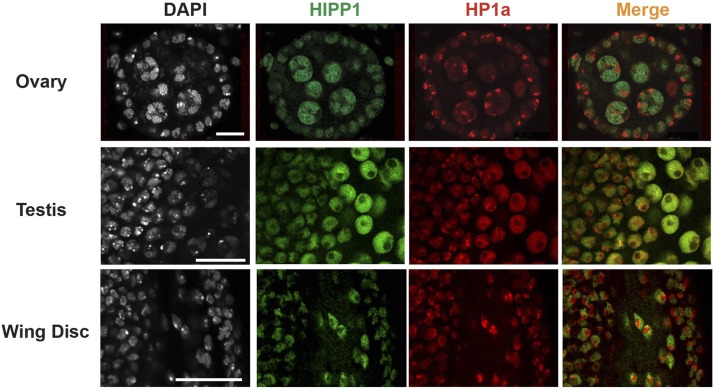
HIPP1 and HP1a show limited co-localization in interphase cells. A. *Top*: Representative confocal image of an early stage egg chamber (stage 4) in an ovary dissected from 1< day-old *Hipp1^GFP/+^* female stained with DAPI, α-GFP (green), and α-HP1a (red). *Middle*: Representative confocal image of the anterior portion of a 1< day-old testis dissected from a *Hipp1^GFP/+^* male and stained as described in A. Scale bars, 20 μm. Anterior is to the left. In testes, HP1a localizes diffusely in spermatocyte nuclei. *Bottom*: Representative confocal image of a third instar larval wing disc stained as described in A.

**Figure 6:**
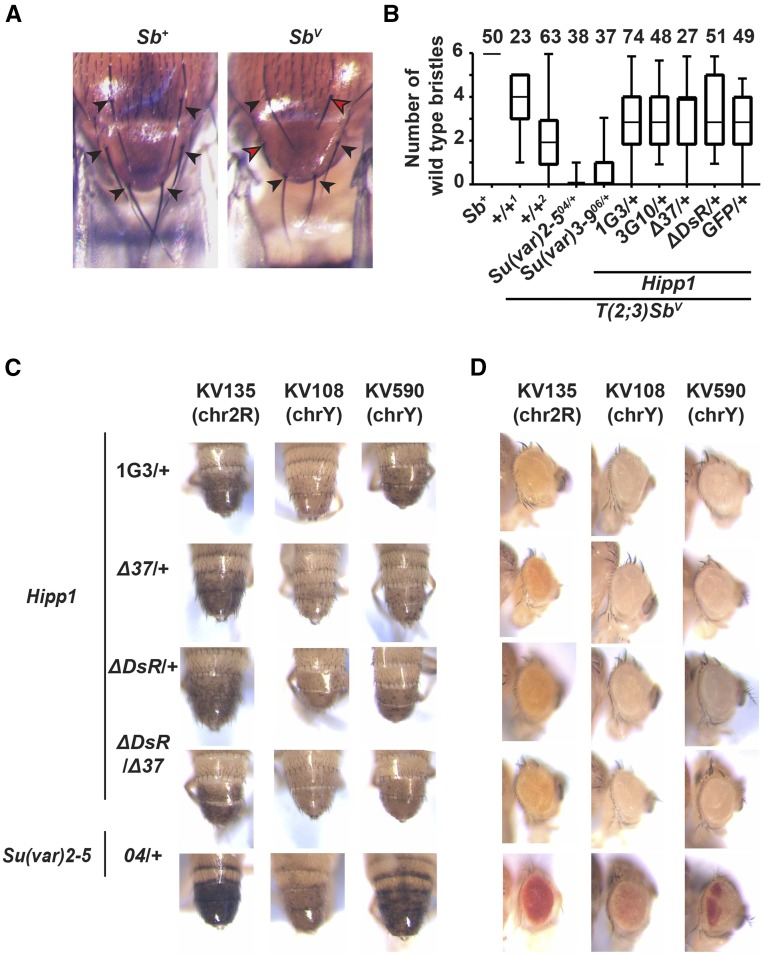
HIPP1 is not essential for HP1a-associated transcriptional silencing. A. Images of the thorax in a *Sb^+^* (*Canton S*) and a *Sb^V^* animal, illustrating the six thoracic bristles that were quantified as either long (black arrowhead) or short (red arrowhead). B. Box plots of quantification of the number of long bristles per thorax of female progeny resulting from crosses of wild type females (Sb*^+/+^*, *Canton S*) or *Sb^v/+^* females with males that have no modifier mutations (1, *Canton S*; 2, *yw*), males with known modifier mutations [*Su(var)2-5^04^*, *Su(var)3-9^06^*], or with *Hipp1* mutant males of the indicated genotypes. The number of individuals scored is shown above each bar. Each box represents the 25^th^ to 75^th^ percentile interval, the line represents the median, and the whiskers represent the range. C. D. Images of abdominal body (C) or eye (D) pigmentation from representative males carrying heterochromatic *SUPorP* insertions crossed into a reference background expressing full length HIPP1 (*Hipp^1G3/+^*), as well as mutant backgrounds heterozygous or homozygous loss of HIPP1 or HP1a [*Su(var)2-5^04^*]. Phenotypes of newly eclosed males were determined. Shown are males represent the median level of pigmentation within a randomly selected collection.

HIPP1 was biochemically identified as a high confidence HP1a interacting protein (Alekseyenko *et al.* 2014). Based on these findings, we predicted that we would detect foci of HIPP1 in our immunohistochemical analyses, but this was not observed (Figure 4). To directly assess the HIPP1 and HP1a partnership, we co-stained *Hipp1^GFP^* derived tissues with antibodies against GFP and HP1a. These two proteins show limited co-localization in cells that carry discrete HP1a loci, including cells in the ovary, testis and wing disc (Figure 5). Despite these findings, genome-wide mapping studies in S2 cells had determined that HIPP1 broadly associated with heterochromatic regions (Alekseyenko *et al.* 2014). In S2 cells, HIPP1 is enriched in *Y*, second and third chromosome heterochromatin, but depleted in *X* chromosome heterochromatin. These observations suggest that HIPP1 might be required for HP1a function in heterochromatic regions outside of the *X* chromosome. To evaluate this postulate, we tested whether *Hipp1* mutants modified transcriptional silencing of reporter genes displaying PEV due to the stochastic spread of heterochromatin. First, we determined whether *Hipp1* mutants modified PEV of the *Sb^1^* allele in the context of *T(2:3)Sb^v^* allele that carries a translocation that places the dominant *Sb^1^* mutation adjacent to centric heterochromatin of the second chromosome. Flies carrying *T(2:3)Sb^v^* display a mosaic thoracic bristle phenotype, wherein bristles are both short (Sb) and long (Sb^+^; Figure 6A). This phenotype reflects the variable spread of heterochromatin into the *Sb^1^* gene. When heterochromatin reaches *Sb^1^*, the dominant mutant allele is inactivated, and a long bristle length is restored. Genetic backgrounds that decrease levels of heterochromatin, such as mutations in the gene encoding HP1a [*Su(var)2-5*] or the histone H3K9 methyl transferase *Su(var)3-9*, dominantly decrease the number of long, wild type bristles (Figure 6B). We crossed *T(2:3)Sb^v^* into multiple *Hipp1* mutant backgrounds, including an allele that generates full length HIPP1 protein lacking two amino acids (negative control, *Hipp1^1G3^*) and three *Hipp1* null alleles (*Hipp1^3G10^*, *Hipp1^Δ37^*, *Hipp1^Δ DsR^*). In each case, the number of Sb^+^ (long, wild type) and Sb (short, mutant) bristles were quantified. We found that the number of long bristles was similar between all *Hipp1* hemizygous offspring (Figure 6B). These data suggest that HIPP1 does not dominantly modify *Sb* variegation. We were unable to test whether complete loss of HIPP1 affected *Sb* variegation, because the *T(2:3)Sb^v^* translocation includes the third chromosome that carries the *Hipp1* gene. As a second test, we determined whether *Hipp1* mutants modified PEV of the *yellow* and *white* genes within *SUPor P* transposons integrated into second or *Y* heterochromatin (Figure 6C, D). We analyzed three *SUPor P* transgenic lines, chosen because these lines displayed moderate to severe repression of *yellow* and *white* gene expression (see unmodified phenotype *Hipp1^1G3/+^*) and were sensitive to HP1a loss (see suppressed phenotype *Su(var)2-5^04^*). Whereas heterozygous loss of HP1a partially restored *yellow* and *white* expression, phenotypic improvement was absent in both heterozygous and homozygous *Hipp1* null mutants (Figure 6C, D). These data indicate that HIPP1 is not essential for HP1a-dependent heterochromatin formation on the second or *Y* chromosomes and are consistent with our findings of limited co-localization with HP1a. Taken together, these studies indicate a limited partnership between HIPP1 and HP1a, a surprising result given their strong association in S2 cells. We suggest that this protein partnership might be regulated. We note that the composition of heterochromatin changes during the cell cycle, demonstrated by the movement of TFs such as GAGA factor or Proliferation disrupter (Prod) between euchromatin and satellite sequences in heterochromatin (Platero *et al.* 1998). In our studies, we primarily examined non-dividing cells, whereas S2 cells are actively dividing. It remains possible that differences in protein composition in heterochromatin during different stages of the cell cycle influences HIPP1 recruitment or stabilization at these genomic regions.

### HIPP1 is not required for fertility

The extensive co-localization of Su(Hw) and HIPP1 in the ovary and testes suggested that HIPP1 might contribute to the function of Su(Hw) in these tissues. For this reason, we determined effects of HIPP1 loss on oogenesis and spermatogenesis, as well as tested genetic interactions between *Hipp1* and *su(Hw)* mutants. In all cases, we examined effects in mutant animals that carried heteroallelic combinations to avoid complications associated with homozygous chromosomes that carry second site mutations.

**Figure 7:**
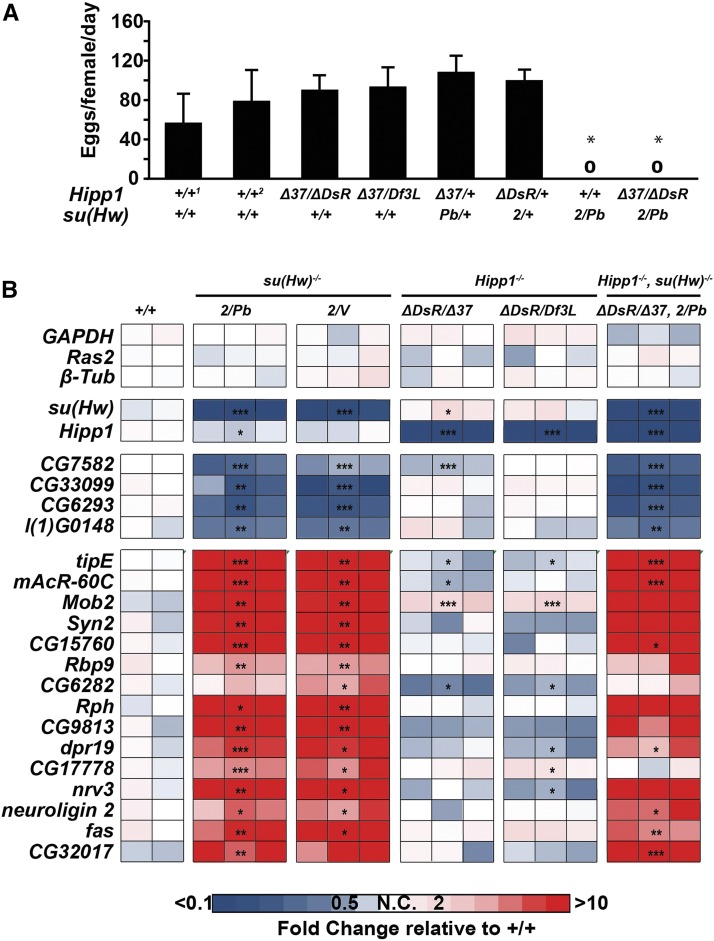
*Hipp1* mutant females are fertile and do not phenocopy Su(Hw) loss. A. Fecundity (eggs laid per female per day) of five-day-old *Hipp1^−/−^*, *su(Hw)^−/−^* and heterozygous *Hipp1^-/+^*, *su(Hw)^-/+^* mutant females of the indicated genotypes, crossed to wild type males. The two wild type (+/+) reference strains were *Canton S* (1) and *yw* (2). Genotypes are noted under the graph. Error bars indicate the standard deviation from a minimum of three independent experiments. Fecundity was compared between genotypes using a one-way ANOVA followed by Tukey *post hoc* analysis. Asterisks indicate genotypes that were significantly different from control lines (p value < 0.01). Only *su(Hw)* null backgrounds showed a significant difference in egg laying. B. Heat map of fold changes of gene expression defined by quantitative reverse transcription PCR (RT-qPCR) of Su(Hw) target genes, measuring gene expression levels in RNA isolated from 1< day-old *su(Hw)^+/+^* (*Canton S*), two *su(Hw)^−/−^*, two *Hipp1^−/−^* and one *Hipp1^−/−^*, *su(Hw) ^−/−^* double mutant backgrounds. Fold change in expression was determined by normalizing levels to the housekeeping gene *RpL32* and is relative to RNA levels in one of the three *su(Hw)^+/+^* (*Canton S*) RNA samples. The color key corresponding to fold change is shown below. Asterisks indicate gene expression changes relative to *Canton S*, * *P* < 0.05. ** *P* < 0.01, *** *P* < 0.001 (Student’s *t*-test).

We determined whether HIPP1 was required for oogenesis. First, we measured the fecundity of *Hipp1^−/−^* females and found that HIPP1 loss did not decrease egg production (Figure 7A). Further, *Hipp1^−/−^* ovaries carried all stages of oogenesis with only low levels of apoptosis of mid-stage egg chambers (Table 2), as defined by staining with antibodies against Vasa and DAPI to reveal apoptotic egg chambers. Second, we tested for genetic interactions between *Hipp1* and *su(Hw)* mutants. Trans-heterozygotes (*Hipp1^Δ37/+^*, *su(Hw)^Pb/+^* and *Hipp1 ^Δ DsR^^/+^*, *su(Hw)^2/+^*), as well as *Hipp1* mutants that were heterozygous for a *su(Hw)* mutation (*HIPP1 ^Δ 37/ ΔDsR^*, *su(Hw)^2/+^*), all showed normal oogenesis and egg production (Table 2, Figure 7A). Third, we determined whether Su(Hw) regulated genes were mis-regulated in *Hipp1* mutant ovaries. We reasoned that loss of HIPP1 might alter transcription of Su(Hw) regulated genes without affecting oogenesis, as our prior studies showed that up-regulation of *Rbp9* was primarily responsible for *su(Hw)^−/−^* infertility (Soshnev *et al.* 2013). To this end, we isolated RNAs from *Hipp1* and *su(Hw)* mutant ovaries and measured RNA levels using qPCR (Figure 7B). In total, the transcriptional output of four Su(Hw) activated and fifteen Su(Hw) repressed genes was defined. We reasoned that if HIPP1 was required for Su(Hw) regulation, then both heteroallelic mutant backgrounds should show changes in gene expression similar to those found in *su(Hw)* mutants. Notably, only one gene met this criteria. *Mob2* was up-regulated in both *Hipp1* mutant backgrounds, although the degree of up-regulation was reduced relative to that found in *su(Hw)* mutants (Figure 7B). Based on these data, we conclude that HIPP1 has minimal contributions to Su(Hw) regulated transcription in the ovary.

**Table 2:** Effects of *Hipp1* mutants on mid-oogenesis egg chamber death

Genotype	% Apoptosis	# ovarioles scored
*Hipp1^+/+^*, *su(Hw)^+/+^ (CS)*	4.7	107
*Hipp1^+/+^*, *su(Hw)^+/+^ (yw)*	1.7	230
*Hipp1^+/+^*, *su(Hw)^Pb/+^*	5.9	85
*Hipp1^+/+^*, *su(Hw)^2/+^*	5.7	140
*Hipp1^+/+^*, *su(Hw)^2/Pb^*	100.0	71
*Hipp1^ΔDsR/+^*, *su(Hw)^2/+^*	0.0	204
*Hipp1^Δ37/+^*, *su(Hw)^Pb/+^*	0.9	217
*Hipp1^ΔDsR/Δ37^*, *su(Hw)^+/+^*	2.0	344
*Hipp1^ΔDsR/Δ37^*, *su(Hw)^2/+^*	6.6	91
*Hipp1^ΔDsR/Δ37^*, *su(Hw)^2/Pb^*	100.0	42

**Figure 8:**
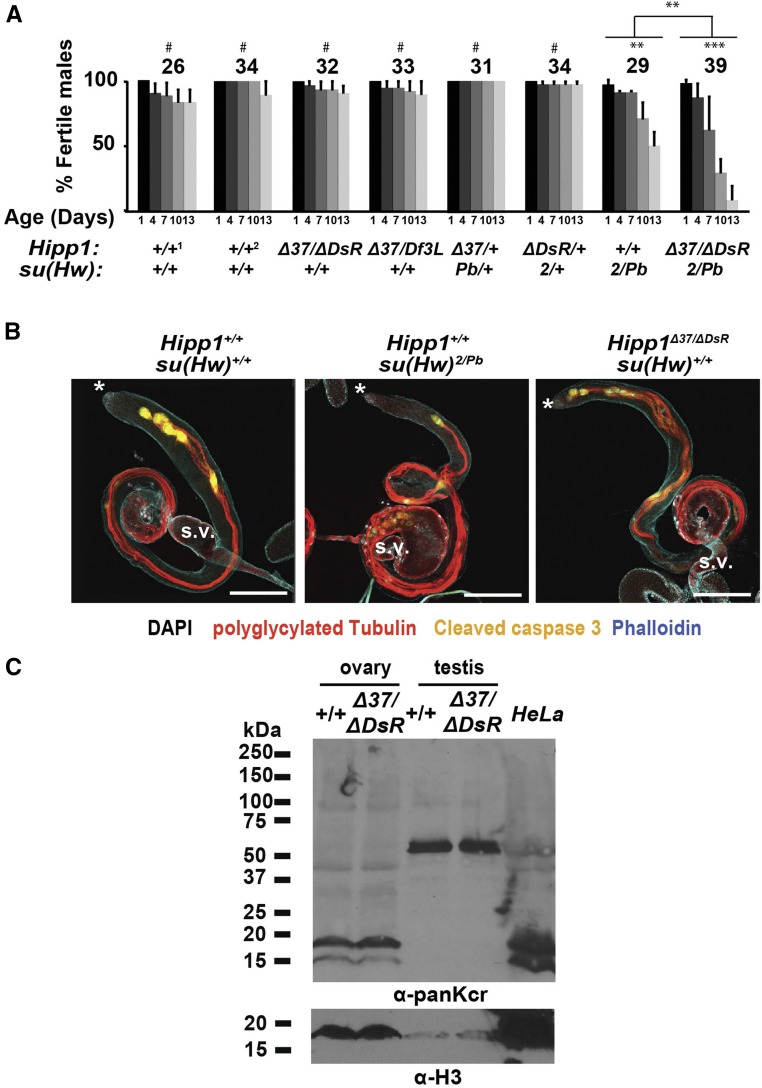
HIPP1 is not essential for spermatogenesis. A. Quantification of the male fertility in wild type (1, *Canton S* and 2, *yw*), two *Hipp1^−/−^*, two heteroallelic *Hipp1^-/+^*, *su(Hw)^-/+^* mutants, and one *Hipp1^−/−^*, *su(Hw) ^−/−^* double mutant background. The number of males tested is shown above each data set. Bars indicate standard deviation from a minimum of three replicates. Significant changes in fertility between groups and over time were determined using repeated-measures ANOVA (#, not significant; ** *P* < 0.01; *** *P* < 0.001). B. Representative confocal images of 3-day-old wild type (*Canton S*), *su(Hw)^2/Pb^* and *Hipp1^Δ37/ΔDsR^* testis stained with antibodies against polyglycylated Tubulin (red, marks sperm tails), cleaved Caspase 3 (yellow, marks ICs) and phalloidin (blue, marks actin in ICs and elsewhere in the testis). Scale bars: 200 μm. Asterisk marks anterior of testis. S.V. denotes the seminal vesicle. C. Western blot of proteins extracted from 1-to 3-day old ovaries and <1 day-old testes from *Hipp1*^+/+^ and *Hipp1^Δ37/ΔDsR^* animals probed with a pan α-crotonyl-lysine (panKcr) antibody and an antibody against Histone H3 (α-H3). A nuclear HeLa cell extract was run as a positive control.

We also determined the requirement for HIPP1 in spermatogenesis. Notably, mouse CDYL has been implicated in spermatogenesis (Lahn *et al.* 2002; Caron *et al.* 2003; Liu *et al.* 2017). Immunohistochemical studies found that CDYL is expressed mainly in round spermatids and spermatocytes (Liu *et al.* 2017), a pattern similar to HIPP1 (Figure 4C). Investigation of the effects of CDYL loss on spermatogenesis is challenging because of the lethality of the *Cdyl* knockout mouse (Wan *et al.* 2013). However, tests of over-expression of CDYL were completed, which showed compromised sperm production, with these defects linked to hypo-crotonylation of histones (Liu *et al.* 2017). First, we measured fertility of *Hipp1^−/−^* males. These studies used a sperm depletion assay to monitor offspring produced as males age (Figure 8A). In contrast to *su(Hw)* mutants, we found that *Hipp1^−/−^* males retained wild type levels of fertility over the two-week period, indicating sustained spermatogenesis. Second, we tested for genetic interactions between *Hipp1* and *su(Hw)* mutants. These studies uncovered a genetic interaction only in *Hipp1^Δ37/ ΔDsR^*, *su(Hw)^2/Pb^* males, wherein fertility of these males declined faster than that of *su(Hw)^2/Pb^* males (Figure 8A). These findings suggest that HIPP1 has modest contributions to male fertility in a *su(Hw)* mutant background. Third, we examined the testis phenotype in *Hipp1* mutant males. We focused on post-meiotic stages of spermatogenesis, because Su(Hw) loss affects these stages (Duan and Geyer 2018). A critical component of these stages is sperm individualization, which is characterized by the formation of actin rich individualization complexes (ICs) around sperm nuclei. IC complexes travel from the posterior to anterior tip of the testis, promoting encasement of each sperm in its own plasma membrane [Figure 8B; (Fabian and Brill 2012)]. As *su(Hw)^−/−^* testes age, ICs become disorganized and a bulge appears at the end of the testes, two defects that coincide with reduced sperm production and small seminal vesicles (Figure 8B). To evaluate *Hipp1^−/−^* testis phenotypes, we stained three-day-old testes with antibodies against polyglycylated Tubulin (polyG Tub) that identifies sperm tails undergoing individualization, as well as phalloidin and antibodies against cleaved Caspase 3 to identify ICs. These analyses reveal a wild type testis phenotype (Figure 8B), with evidence of continued IC formation, the absence of a posterior bulge and large seminal vesicles. The lack of a shared mutant phenotype between *su(Hw)* and *Hipp1* null males suggests that HIPP1 is not required for transcription of Su(Hw) regulated genes involved in spermiogenesis. Fourth, we examined histone crotonylation in the *Hipp1^−/−^* testes, as CDYL has been implicated in the negative regulation of histone lysine crotonylation (Kcr) in spermatogenesis (Liu *et al.* 2017). If HIPP1 functions similarly to CDYL in the testes, then levels of histone Kcr should increase. To this end, western analysis was completed with the α-crotonyl-lysine (panKcr) antibody used previously (Liu *et al.* 2017). Only low levels of histones were recovered in testes extracts, with these histones showing low levels of Kcr that was unchanged upon HIPP1 loss (Figure 8C; Fig. S2). Instead, the major testis Kcr protein is an ∼50 kD protein, with the level of Kcr modification unchanged in *Hipp1* mutants. We also analyzed protein extracts obtained from wild type and *Hipp1^−/−^* ovaries. In this case, histone Kcr is abundant, and is unchanged upon HIPP1 loss (Figure 8C). These observations suggest that loss of HIPP1 does not affect levels of crotonyl-lysine in germ cells. Taken together, we conclude that HIPP1 is not essential for spermatogenesis.

### Su(Hw) recruits HIPP1 to euchromatic regions

**Figure 9:**
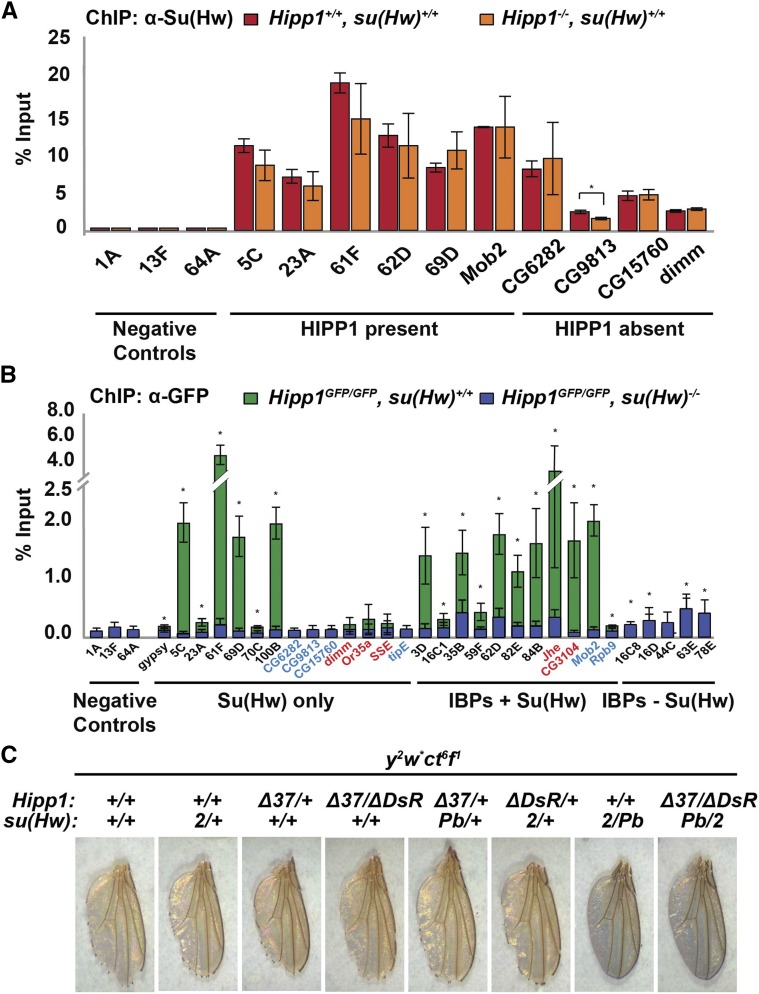
HIPP1 occupancy at euchromatic sites depends on Su(Hw). A. ChIP-qPCR analysis of Su(Hw) binding in *Hipp1^−/−^* (orange) and wild type (*Canton S*, red) ovaries. Three classes of sites were tested, including sites that lack Su(Hw) and HIPP1 (negative controls), SBSs that bind HIPP1 (HIPP1 present), and SBSs that lack HIPP1 (HIPP1 absent). Bars represent standard deviation of at least two biological replicates. Asterisks indicate a significant change in Su(Hw) binding between genotypes (*t*-test, p-value <0.05). B. ChIP-qPCR analysis of HIPP1-GFP occupancy in the ovary, using chromatin isolated from *Hipp1^GFP/GFP^*, *su(Hw)^+/+^* females (green) or *Hipp1^GFP/GFP^*, *su(Hw)^2/Pb^* mutant (blue) background. Four classes of sites were tested based on HIPP1 occupancy in S2 cells (Alekseyenko *et al.* 2014), including 1) negative controls that lack HIPP1, Su(Hw) and other IBPs, 2) Su(Hw) only SBSs, 3) Su(Hw) SBSs bound by other IBPs, 4) IBP that lack Su(Hw). Tested sites were named for their cytological position or for the Su(Hw) target gene that are mis-regulated in *su(Hw)^−/−^* ovaries (blue) or testes (red). Asterisks indicate a significant enrichment of HIPP1-GFP in *su(Hw)^+/+^* relative to the negative controls, p-value <0.05 (*t*-test). Bars represent the standard deviation from three independent replicates. C. Shown are representative wings dissected from 1< day-old males that carry an *X* chromosome with two classic mutations caused by the *gypsy* insulator, *yellow^2^* (*y^2^*) and *cut^6^* (*ct^6^*). Males were generated that carried the *y^2^ ct^6^* chromosome and were *Hipp1^−/−^*, heterozygous *Hipp1^-/+^*, *su(Hw)^-/+^* or *Hipp1^−/−^*, *su(Hw)^−/−^*, as indicated. When insulator function is lost, males carrying the *y^2^ ct^6^* chromosome have a dark wing blade and smooth wing margin.

Chromosome association of Su(Hw) is influenced by its cofactor Mod67.2 (Soshnev *et al.* 2012). We wondered whether HIPP1 also facilitated Su(Hw) chromosome association. To answer this question, we completed ChIP-qPCR of Su(Hw) in a *Hipp1* null background. These studies found Su(Hw) binding to SBSs was unchanged upon HIPP1 loss (Figure 9A). We conclude that HIPP1 is not required for Su(Hw) binding in the genome.

Although our data suggest that HIPP1 is not essential for transcription of Su(Hw) regulated genes, over half of HIPP1 associated regions are bound by Su(Hw) (Alekseyenko *et al.* 2014). This prompted our investigation of whether Su(Hw) is required for recruitment of HIPP1 to euchromatin. First, we identified HIPP1 occupied sites in ovary. We dissected ovaries from *Hipp1^GFP^* females and immunoprecipitated HIPP1 using GFP antibodies, analyzing DNA enrichment using qPCR (ChIP-qPCR). Parallel studies were conducted using Canton-S ovaries as a negative control, confirming the specificity of the GFP antibodies (data not shown). In total, 30 genomic regions were assayed. We chose these regions based on two criteria. First, HIPP1 occupied these sites in S2 cells. Second, these regions were occupied by different IBPs. The categories included Su(Hw) and HIPP1 absent regions (negative controls), as well as HIPP1 regions that were SBSs bound only by Su(Hw) [Su(Hw) only], SBSs bound by other IBPs [Su(Hw)+ IBPs] and non-Su(Hw) IBPs [IBPs-Su(Hw); Table S5]. We found HIPP1-GFP associated with 73% (22/30) of the predicted HIPP1 regions, including 72% (18/25) of all SBSs and 80% (4/5) of non-Su(Hw) IBPs regions (Figure 9B). These data suggest that the overlap of HIPP1 occupancy in S2 cells and the ovary is strong. Strikingly, the level of HIPP1-GFP association with SBSs is higher than at non-Su(Hw) IBP regions. Even so, many SBSs did not display HIPP1 association. Notably, the majority of these ovary lost regions (88%, 7/8) correspond to SBSs in Su(Hw) regulated genes. These findings are consistent with our observations that HIPP1 is not required for Su(Hw)-dependent transcription in the ovary.

Having identified ovarian HIPP1 occupied regions, we asked whether Su(Hw) played a role in HIPP1 chromosome association. We reasoned that if Su(Hw) were required for HIPP1 recruitment and/or retention, then loss of Su(Hw) would reduce HIPP1 association at SBSs and not at other genomic regions. To this end, we conducted ChIP-qPCR analysis of chromatin isolated from *Hipp1^GFP/GFP^*, *su(Hw)^−/−^* females. Indeed, loss of Su(Hw) significantly decreased the level of HIPP1-GFP association at the vast majority (94%; 17/18) of SBSs, whereas retention at all non-Su(Hw) regions was not affected (4/4, Figure 9B). Strikingly, the level of HIPP1 association was significantly decreased at regions bound by both Su(Hw) and other IBPs. As SBSs represent a large component (56%) of HIPP1 bound regions, these findings imply that Su(Hw) has a major role in determining HIPP1 euchromatic occupancy.

HIPP1 binds the *gypsy* insulator in the ovary (Figure 9B). These observations suggested that HIPP1 might have a role in establishing *gypsy* insulator function. To test this possibility, we defined effects of HIPP1 loss on enhancer blocking, using the classic *gypsy*-induced *yellow^2^* and *cut^6^* mutations (Corces and Geyer 1991). The *gypsy* insulator in the *y^2^* gene blocks the action of the wing and body enhancers, producing a yellow wing blade and light color body. The *gypsy* insulator in the *ct^6^* gene blocks the wing margin enhancer, causing cuts or notches in the wing margin. All of these mutant phenotypes are reversed by loss of Su(Hw) [Figure 9C, *su(Hw)^2/Pb^*]. To access effects of HIPP1 loss on gypsy insulator function, we assessed wing phenotypes. In *y^2^ ct^6^*, *su(Hw)^2/Pb^* mutants, the wings are dark and smooth (Figure 9C). In contrast, *y^2^ ct^6^*, *Hipp1^Δ37/ΔDsR^* mutants had yellow and notched wings, indicating that the *gypsy* insulator remains functional. Further, *Hipp1* mutants failed to dominantly enhance *gypsy*-induced mutant phenotypes found in heterozygous *su(Hw)* mutants (Figure 9C). These studies reveal that HIPP1 is not required for enhancer blocking by the *gypsy* insulator.

### Concluding remarks

HIPP1 is a biochemically identified partner protein of HP1a and Su(Hw) (Alekseyenko *et al.* 2014; Rhee *et al.* 2014). Here, we investigated functional contributions of HIPP1 to Drosophila development. We generated multiple *Hipp1* null alleles (Figure 3), finding that *Hipp1* is a non-essential gene that is dispensable for female and male fertility (Table 1, Figures 5,6). In interphase cells, we show that HIPP1 is a broadly expressed nuclear protein that largely localizes to chromosomes outside of heterochromatic domains (Figure 4, Fig. S1A). These observations prompted investigation of HIPP1 contributions to heterochromatin formation, testing whether loss of HIPP1 alters HP1a-dependent transcriptional silencing of three different reporter genes inserted into HIPP1 and HP1a-enriched heterochromatic regions (Alekseyenko *et al.* 2014). Strikingly, neither heterozygous or homozygous loss HIPP1 reversed transcriptional silencing of reporter genes experiencing HP1a-dependent PEV (Figure 6), indicating that HIPP1 has a non-essential partnership with HP1a in heterochromatin. We also investigated a euchromatic role for HIPP1. Our data support earlier findings that Su(Hw) and HIPP1 are protein partners (Alekseyenko *et al.* 2014; Rhee *et al.* 2014). We find that HIPP1 chromosome association in the ovary strongly overlaps that defined in S2 cells (Figure 9B). Further, HIPP1 occupancy is highest at SBSs relative to other IBP sites and depends upon Su(Hw) (Figure 9B). Even so, HIPP1 is not required for Su(Hw) regulatory functions, including its repressor, activator or insulator functions (Figures 7, 9). Notably, the absence of a regulatory contribution correlates with low HIPP1 occupancy at Su(Hw) regulated gene SBSs and the *gypsy* insulator (Figure 9B).

Although HIPP1 is not essential for Drosophila development, mouse CDYL is required for viability and is linked to male fertility (Wan *et al.* 2013; Liu *et al.* 2017). Homology between these proteins is restricted to the carboxyl-terminal crotonase-like fold domain, a domain with demonstrated crotonyl CoA hydratase function (Liu *et al.* 2017). These observations raise the possibility that other crotonase domain proteins in Drosophila compensate for HIPP1 loss during development. Indeed, the Drosophila genome encodes seventeen proteins with predicted crotonase-like fold domains, seven with significant homology to HIPP1 (*CG4594*, *CG5844*, *CG6543*, *CG6984*, *CG8778*, *CG9577* and *CG13890*). Analysis of the exon-intron structure within the crotonase-encoding regions of this subset of genes supports possible ancestry only between *Cdyl* and *Hipp1* or *CG13960*. Our analyses showed that the *Hipp1* and *Cdyl* genes share three splice junctions, whereas *CG13960* and *Cdyl* share one. However, the CG13960 crotonase domain displays stronger homology with Peroxisomal 3,2-*trans*-enoyl-CoA isomerase (PECI) than CDYL. Additionally, only HIPP1 co-purified with HP1a or Su(Hw), with no other crotonase-fold protein showing significant association (Alekseyenko *et al.* 2014; Rhee *et al.* 2014). Taken together, these findings indicate that HIPP1 is the fly crotonase domain protein that represents the homolog of CDYL. As such, our data suggest that the human and fly homologs have evolved different developmental roles. Whereas CDYL has an essential developmental role, HIPP1 does not. Further studies are needed to resolve how HIPP1 contributions to fly development.
